# The Presence of Psoriasis, Metabolic Syndrome and Their Combination Increases the Serum Levels of CRP and CD5L but Not sCD200R1 and sTLR2 in Participants

**DOI:** 10.3390/jpm12121965

**Published:** 2022-11-28

**Authors:** Drahomira Holmannova, Pavel Borsky, Ctirad Andrys, Jan Krejsek, Eva Cermakova, Zdenek Fiala, Kvetoslava Hamakova, Tereza Svadlakova, Helena Parova, Vit Rehacek, Gabriela Poctova, Lenka Borska

**Affiliations:** 1 Institute of Preventive Medicine, Faculty of Medicine in Hradec Kralove, Charles University, 500 03 Hradec Kralove, Czech Republic; 2 Institute of Clinical Immunology and Allergology, University Hospital, Faculty of Medicine in Hradec Kralove, Charles University, 500 03 Hradec Kralove, Czech Republic; 3 Institute of Medical Biophysics, Faculty of Medicine in Hradec Kralove, Charles University, 500 03 Hradec Kralove, Czech Republic; 4 Clinic of Dermal and Venereal Diseases, University Hospital Hradec Kralove, 500 03 Hradec Kralove, Czech Republic; 5 Institute of Clinical Biochemistry and Diagnostics, University Hospital, Faculty of Medicine in Hradec Kralove, Charles University, 500 03 Hradec Kralove, Czech Republic; 6 Transfusion Center, University Hospital, 500 03 Hradec Kralove, Czech Republic

**Keywords:** psoriasis, sCD200R1, CD5L, sTLR2, metabolic syndrome

## Abstract

Psoriasis and metabolic syndrome (MetS) are chronic inflammatory conditions associated with the dysregulation of immune system reactivity. The inflammatory processes of both diseases have not yet been fully characterized, and the evaluation of proteins/markers that could be involved in their pathogenesis is of great importance. We selected four markers: CRP, sCD200R1, CD5L, and sTLR2; in particular, sCDR2001 has not yet been measured in the context of psoriasis and metabolic syndrome. **Material and methods:** In the study, 64 controls and 43 patients with psoriasis with or without a metabolic syndrome were enrolled. The levels of selected markers were measured using ELISA kits. **Results:** CRP levels were significantly higher in psoriasis patients, especially in the subgroup of patients with MetS compared to nonMetS patients (*p* < 0.01). sCD200R1 and sTLR2 were not significantly different between groups and subgroups; however, CD200R1 levels were slightly higher in both control groups compared to both groups of patients. CD5L levels were significantly higher in patients with MetS compared to nonMets patients (*p* < 0.02). We also evaluated the correlations between parameters in controls and patients’ groups, as well as in subgroups. Correlations between BMI and CRP were found in all groups and subgroups. Other correlations were group- and subgroup-specific. For example, in the patients’ group, CD5L correlated with sCD200R1 (*p* < 0.05) and in MetS controls, with age (*p* < 0.03). **Conclusion:** The results show that the presence of systemic inflammation associated with psoriasis and metabolic syndrome and their combination alters the expression of specific molecules, especially CRP and CD5L, which were significantly increased in patients with psoriasis and a metabolic syndrome compared to controls without metabolic syndromes. Correlations between CRP and BMI in all groups suggest that overweight and obesity increase the intensity of inflammation and potentiate CD5L expression. In contrast, levels of molecules that may limit inflammation were not increased in psoriasis and metabolic syndrome subjects (they were non-significantly lower compared with healthy controls), which may reflect the chronic nature of both diseases and the exhaustion of inhibitory mechanisms.

## 1. Introduction

Psoriasis has long been considered a disease localized on the skin, and nails, or in the joints, in the case of psoriatic arthritis. Today, it is clear that psoriasis is a chronic systemic inflammatory disease that negatively affects the entire organism [1]. Genetic factors are involved in the development of psoriasis, and predispose people with psoriasis to a dysregulated immune response to triggering factors, such as infections, medications, skin injury, and stress [2,3]. The clinical presentation, distribution, and severity of psoriasis are highly variable. Cutaneous manifestations of psoriasis include the presence of psoriatic lesions characterized by red, scaly papules and plaques covered with dry gray or silver scales. These skin changes are associated with abnormal differentiation and hyperproliferation of keratinocytes, infiltration of the skin by immune cells, inflammation, and neoangiogenesis. Typically, lesions occur on the knees, elbows, scalp, and lumbosacral region [4].

The chronic inflammatory process is a risk factor for the development of a wide variety of diseases and comorbidities. People with psoriasis are more likely to suffer from other autoimmune, neurodegenerative, psychiatric, or metabolic diseases compared to the healthy population. One of the most common complications is metabolic syndrome [5,6].

Metabolic syndrome, as well as psoriasis, is characterized by systemic chronic inflammatory disease that is diagnosed based on the following criteria: increased triglycerides, fasting glucose, waist circumference, blood pressure, and a decrease in HDL levels [7,8]. The presence of adipose tissue hypertrophy and adiposopathy, insulin and leptin resistance, gut dysbiosis, and free fatty acids are responsible for the dysregulation of immune system activity and inflammation, especially in blood vessels, adipose tissue, the liver, the pancreas, and the central nervous system [9,10]. The combination of both diseases has a detrimental impact on health and quality of life. Our previous studies show that the combination of both diseases increases CRP, calprotectin and angiopoietin-like 8 levels, DNA damage, and chromosomal aberrations compared to patients with either psoriasis or a metabolic syndrome alone. This may indicate, among other things, an increased risk of cancer, other inflammatory and neurodegenerative diseases, etc. [11,12,13].

We selected markers that may be involved and modulate inflammation in both pathologies.

CD200R1 is an inhibitory receptor that belongs to the immunoglobulin family, with restricted expression to immune cells. Its expression is increased in the inflammatory microenvironment. Inhibitory signaling after CD200 ligation depends on three tyrosine residues that act as an ITIM domain on other inhibitory receptors. The main targets of CD200R1 activation are Ras, ERK, and p38MAPK [14]. The soluble form of the receptor is produced by alternative mRNA splicing or cleavage of the receptor expressed on membranes by metalloproteinases. While the bound form of CD200R1 suppresses inflammation, the soluble form lacks the ability to induce intracellular signaling pathways, leading to inhibition of inflammation binds to CD200 and sCD200, and blocking its interaction with the membrane CD200R1 [15]. Their anti-inflammatory/homeostatic potential is lost in this way.

CD5-Like (CD5L) is a pleiotropic secreted protein that belongs to the cysteine-rich scavenger receptor family, which is synthesized mainly by macrophages. CD5L is a ligand for membrane CD36. CD5L captures PAMP and DAMP, including ox LDL, and promotes their phagocytosis, inhibits apoptosis, promotes cell survival, and modulates and regulates the inflammatory response and lipid metabolism, maintaining tissue homeostasis [16,17]. CD5L has both pro- and anti-inflammatory effects. CD5L influences the Th17 subset function. The subset of Th17 T cells in autoimmune diseases expresses less CD5L, whereas non-pathogenic Th17 cells express a higher amount of this molecule [18]. The decrease in CD5L expression in Th17 turns them into pathogenic cells. In macrophages, CD5L inhibits TNFα and IL-1β production and promotes their differentiation into the M2 subset [19,20]. The interaction of CD5L/oxLDL with CD36 results in its internalization. In macrophages, it causes foamy cell formation, and in adipocytes, it induces lipolysis and lower saturated fatty acid synthesis by binding to fatty acid synthase. This leads to the efflux of free fatty acids from adipocytes, which can interact with TLR4, and decreases the volume of adipose tissue volume and body weight [21,22].

TLR2 is a member of the Toll-like receptor family. It is widely expressed, especially in immune cells, and plays a crucial role in innate immunity. TLR2 recognizes PAMPs and DAMPs (glycolipids, lipopeptides, porins, glycoproteins, and hemagglutinins). Ligation induces the MyD88—IRAK—NF-κB pathway and the production of pro-inflammatory cytokines [23]. The soluble form of TLR2 is formed by post-translational modification or cleavage of the membrane receptor by the metalloproteinases ADAMs [24]. Soluble forms act as a decoy for the TLR2 receptor and inhibit the inflammatory response [25].

Our selected markers could reflect both pro-inflammatory and anti-inflammatory reactions associated with psoriasis and metabolic syndrome.

## 2. Materials and Methods

### 2.1. Study Groups

A total of 107 individuals were enrolled in our study: 64 controls without psoriasis and 43 patients with psoriasis. Controls and patients were divided into two subgroups due to the presence of metabolic syndrome. The control group included 15 participants with MetS and 49 participants without Mets (nonMetS). The patient group included 15 participants with MetS and 28 nonMetS participants. All subjects (107 individuals) signed an informed consent form before participating in the study. The patients with psoriasis (43 individuals) were examined with blood sampling at the Department of Dermatology, Charles University, Faculty of Medicine and University Hospital in Hradec Kralove. Participants from the control group (64 individuals) were blood donors from the Department of Transfusion Medicine, Charles University, Faculty of Medicine and University Hospital in Hradec Kralove. Exclusion criteria included the presence of any inflammatory diseases, pregnancy, cancer, non-steroidal and anti-inflammatory medications, and treatment of psoriasis fewer than 3 months previously. The study was conducted in accordance with the Declaration of Helsinki, and the protocol was approved by the Ethics Committee of the University Hospital in Hradec Kralove, Czech Republic (project identification code: PROGRES Q40-09, Q40-10, and Q40-11; reference number: 201705 183P; date of approval: 2 May 2017). Informed written consent was obtained from all persons.

### 2.2. PASI

The severity of psoriasis was evaluated using the PASI (Psoriasis Area Severity Index), which is calculated based on erythema, desquamation, induration, and the area of skin involved [26].

### 2.3. Metabolic Syndrome

The presence of metabolic syndrome was evaluated according to the criteria of the National Cholesterol Education Program Adult Treatment Panel (NCE/ATPIII). In order to confirm metabolic syndrome, three or more of these criteria had to be met [27]: (1) increased waist circumference (abdominal obesity) (102 cm for men and ≥88 cm for women); (2) glucose intolerance with higher fasting glucose (5.6 mmol/L) or known treatment for diabetes; (3) increased triglyceride level (TAG) 1.7 mmol/L; (4) reduced level of high-density lipoproteins (HDL cholesterol) (<1.03 mmol/L for men and <1.30 mmol/L for women); (5) elevated systolic blood pressure (130 mmHg) and/or diastolic blood pressure (85 mmHg).

Waist circumference, systolic and diastolic blood pressure, and the PASI were evaluated at the Department of Dermatology, Charles University, Faculty of Medicine and University Hospital in Hradec Kralove. 

### 2.4. Blood Sampling

For the evaluation of the selected markers in all participants, peripheral blood samples were collected from the cubital vein by BD Vacutainer sampling tubes. Blood serum samples were isolated by centrifugation and stored at −70 °C until analysis. Repeated thawing and freezing were avoided.

### 2.5. Levels of sCR200R1

CD200R1 levels were determined using an ELISA kit: ELISA Assay Kit for CD200 Receptor 1 (Cloud Clone Corp., Katy, TX, USA) according to the manufacturer’s instructions. Samples were diluted 100-fold. The detection range was 7.8–1000 pg/mL. The absorbance values were read at 450 nm on a Multiskan RC ELISA reader (Thermo Fisher Scientific, Waltham, MA, USA).

### 2.6. Levels of CD5L

Levels of CD5L were measured using ELISA kit: Human apoptosis inhibitor of macrophage (CD5L) ELISA (BioVendor, Brno, Czech Republic). Samples were diluted 500-fold. The detection range was 0.08–10 ng/mL. The absorbance values were read at 450 nm on a Multiskan RC ELISA reader (Thermo Fisher Scientific, Waltham, MA, USA).

### 2.7. Levels of sTLR2

Levels of sTLR2 were evaluated using an ELISA kit: ELISA Assay Kit For Human TLR-2 (Cloud Clone Corp., USA). The samples were not diluted. The detection range was 0.16–20 ng/mL. The absorbance values were read at 450 nm on a Multiskan RC ELISA reader (Thermo Fisher Scientific, Waltham, MA, USA).

### 2.8. Levels of CRP

CRP levels were determined using IMMAGE CRP Reagent (Beckman Coulter Inc., Pasadena, CA, USA). Samples were diluted 36-fold and read on Immunonefelometr IMMAGE 800 (Beckman Coulter Inc., Pasadena, CA, USA). The detection range was 0.1–80 mg/L.

### 2.9. Statistical Analysis

Quantitative data are presented by median, first, and third quartiles, with a minimum and maximum. Two-sample t-test, as well as nonparametric Mann–Whitney and Kolmogorov–Smirnov tests were used to compare groups. Nonparametric Kruskal–Wallis One-Way Analysis of Variance with post-hoc Dunn’s test and Bonferroni modification was used to compare the four groups. Spearman rank correlations were used to evaluate the relationship between parameters. Qualitative data are presented as counts and percentage, for which Fisher’s exact test was used. Data are graphically presented by box and scatter plots. A level of significance of α = 0.05 was considered statistically significant. Statistical analysis was performed using statistical software NCSS 2021 Statistical Software (2021). NCSS, LLC., Kaysville, UT, USA, ncss.com/software/ncss (accessed on 24 March 2022).

## 3. Results

A total of 43 patients with psoriasis, with or without metabolic syndrome (MetS n = 15 and nonMetS n = 28), and 64 controls, with or without metabolic syndrome (MetS n = 15 and nonMetS n = 49), were enrolled into our study. The distribution of age and sex did not differ significantly between the groups. There was a higher number of smokers in the patient group (Table 1). The average BMI was higher in patients compared to controls (*p* < 0.005), and higher in MetS patients compared to nonMetS patients and MetS controls (*p* < 0.001 and *p* < 0.005) (Table 2). The severity of psoriasis PASI did not differ between MetS and nonMetS patients (Table 3).

### 3.1. Levels of CRP

**Figure 1:**
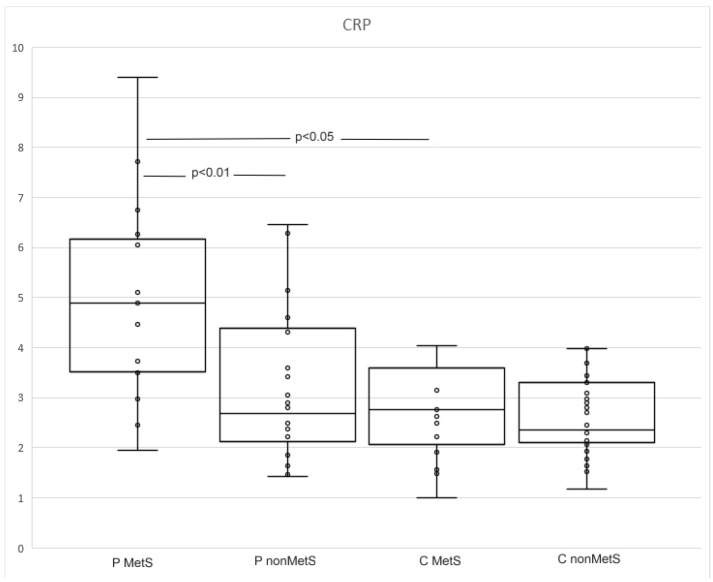
The levels of CRP in MetS and nonMets patients and controls. Within each box, the middle line of the box represents the median. Boxes extend from the 25th–75th percentile of each group’s distribution of values. Whiskers above and below the box indicate the 10th and 90th percentiles.

CRP levels were higher in patients compared to controls (median 2.48, interquartile range 2.1–3.41 and 3.43, 2.28–5.14, *p* < 0.05; Table 4). A subgroup analysis showed that patients with MetS had higher levels of CRP compared to nonMetS patients and both groups of controls (4.96, 3.49–6.27 and 2.68, 1.95–4.63; *p* < 0.01 and *p* < 0.05; Table 5, Figure 1).

### 3.2. Levels of sCD200R1

**Figure 2:**
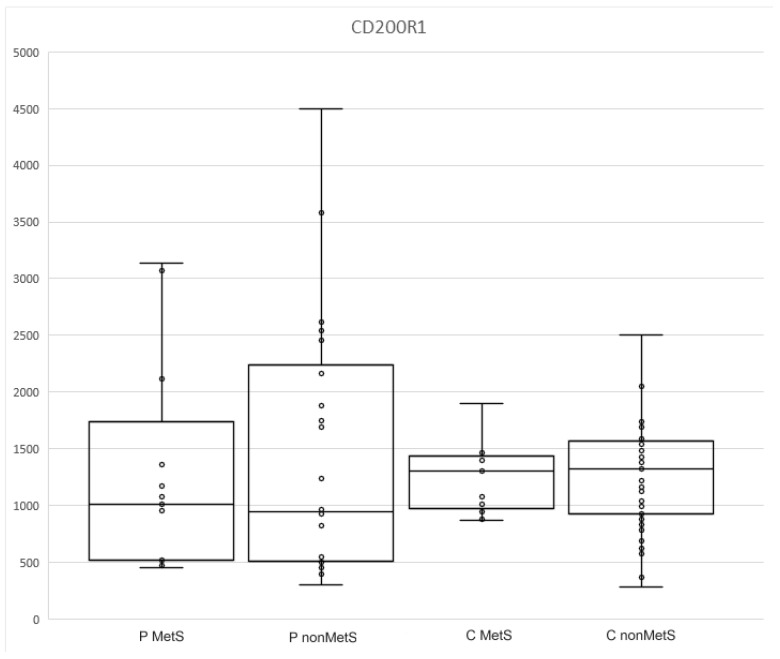
The levels of sCD200R1 in MetS and nonMets patients and controls.

The measured values of CD200R1 did not differ between controls and patients, nor between the subgroups (Table 4 and Table 5, Figure 2). They were slightly higher in both control groups compared to the patient groups (1314.5, 945–1567 and 966, 506–2164; Table 4).

### 3.3. Levels of CD5L

**Figure 3:**
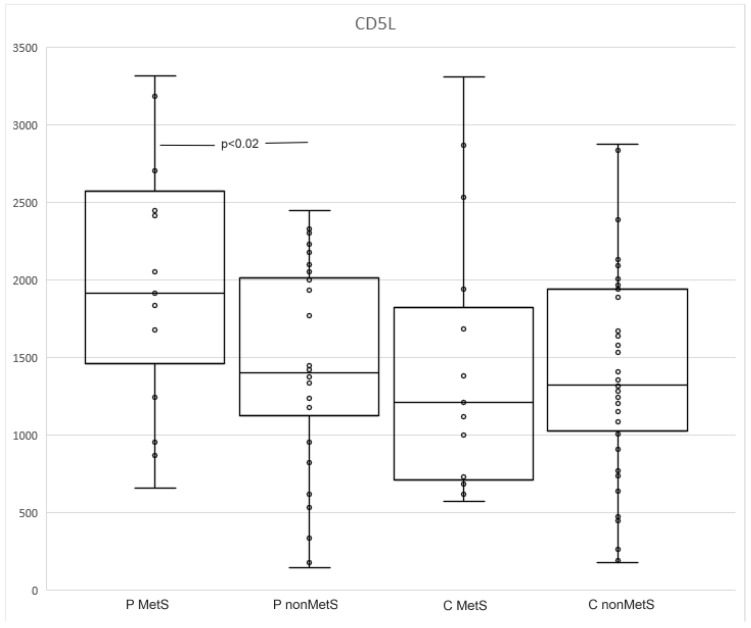
The levels of CD5L in MetS and nonMets patients and controls.

There was no statistical significance between patients and controls in CD5L levels (Table 4). Subgroup analysis revealed that the levels were elevated in patients with MetS compared to nonMets (1915, 1244–2704 and 1400.5, 1008.5–2040; *p* < 0.02; Table 5, Figure 3). We also detected a trend toward significance between the CD5L levels in MetS controls and MetS patients (1207.1, 686–1937.2 and 1915, 1244–2704; *p* < 0.08) (Table 5, Figure 3).

### 3.4. Levels of sTLR2

**Figure 4:**
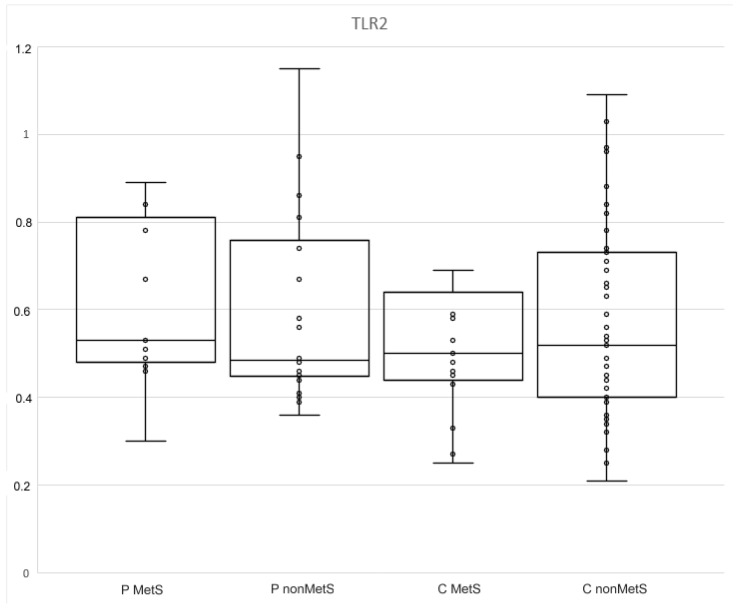
The levels of sTLR2 in MetS and nonMets patients and controls.

The differences between the levels of TLR2 in controls and patients and in all subgroups were not statistically significant (Table 4 and Table 5; Figure 4).

### 3.5. Relationships among Selected Parameters

In the group of patients, the negative correlations between CD5L and CD200R1 and between PASI and age, and positive correlations between CRP and BMI, CD5L and BMI, and PASI and age were identified (Spearman correlation −0.39, *p* < 0.05; SC: −0.3, *p* < 0.05; SC: 0.58, *p* < 0.002; SC: 0.37, *p* < 0.01).

The only correlation in the controls was CRP and BMI (SC: 0.26, *p* < 0.02).

The subgroup analysis revealed two correlations in controls and three in patients. The correlation between CRP and BMI in nonMetS controls, and between CD5L and age in MetS controls (SC: 0.33, *p* < 0.02; C MetS SC: 0.48, *p* < 0.03), were detected. 

**Figure 5:**
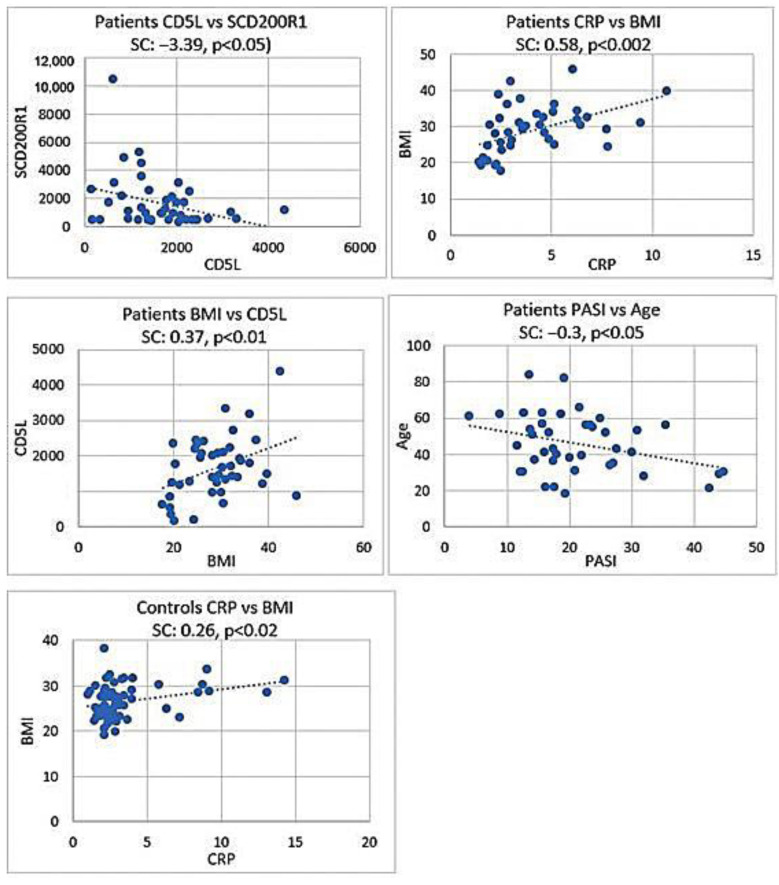

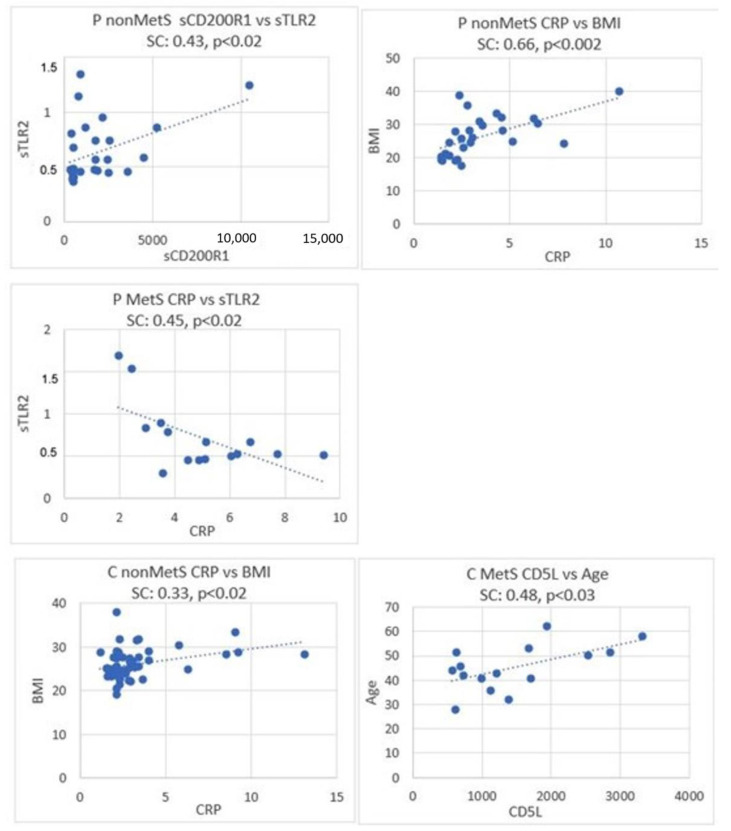
Scatter plots illustrating the correlations between measured parameters, both in groups of controls and patients and in subgroups. Dots represent the values of measured markers of each participant.

There were correlations between CD200R1 and TLR2, and between BMI and CRP, in nonMetS patients (SC:0.43, *p* < 0.02; SC: 0.66, *p* < 0.002). In MetS patients, a negative correlation between CRP and TLR2 was found (SC: −0.45, *p* < 0.02) (Figure 5).

## 4. Discussion

Psoriasis and metabolic syndromes are accompanied by systemic inflammation of varying intensities. Systemic inflammation increases the risk of developing other diseases, including degenerative and cancerous diseases. It is important to explore markers that reflect the inflammatory processes in both diseases, including their progression and risk of complications, and can be used in clinical practice. In our previous studies, we have evaluated other types of markers, e.g., elafin, clusterin, and calprotectin, and in this study we are expanding the spectrum of markers.

As expected, CRP levels (a highly nonspecific marker of inflammation) were significantly higher in MetS patients compared to other subgroups in our study, although they did not reach values that reflect acute inflammation. These results are consistent with a wide range of studies that evaluated CRP in patients with psoriasis and metabolic syndromes [28,29]. We also found that CRP levels were correlated with BMI both in patients and controls. The highest correlation was found among nonMetS patients. There are many studies documenting the relationship between CRP and BMI [30]. Surprisingly, a correlation between CRP and PASI was not found. PASI was negatively correlated with the age of the patients. 

sCD200R1 has not yet been evaluated in patients with psoriasis or metabolic syndromes. Psoriasis and metabolic syndromes are inflammatory conditions that can increase the expression and secretion of sCD200R1. Inflammation increases the expression of both CD200R1 and the activity of metalloproteinases that can cleave membrane CD200R1 and form sCD200R1. Although the data are not statistically significant, surprisingly, sCD200R1 levels were higher in the controls.

The results of other studies have suggested that the increase in CD200R1 expression is possibly related mainly to acute inflammation (e.g., neuroinflammation after stroke, reaction after cardiac surgery), while chronic inflammation is associated with decreased expression of CD200R1, which, in turn, allows the maintenance of inflammatory processes due to alterations of compensatory mechanisms [31,32,33]. The decrease in CD200R1 expression has been documented in SLE, rheumatoid arthritis, inflammatory bowel disease, etc. [34,35,36]. The same decrease has also been documented in patients with obesity and psoriasis. Bories et al. focused on CD200R1 expression in patients with obesity. Its expression was lower in obese patients compared to lean individuals [37]. However, Ismail et al. described that sCD200 levels were elevated in patients with psoriasis, while the expression of CD200R1 was decreased compared to healthy controls [38]. Linley et al. demonstrated that decreased CD200R1 signaling is associated with induction of neutrophil recruitment to the skin in psoriasis [39].

Lower levels of sCD200R1 in our patients may be due to the documented reduced expression of CD200R1, but we must also consider enhanced metalloproteinases activity in psoriasis and metabolic syndromes, which increases membrane CD200R1 shedding. This could elevate sCD200R1 levels, despite its reduced expression; therefore, we might speculate that the decrease in CD200R1 is immunobiologically significant [40,41]. We cannot omit the possibility that levels of sCD200R1 could be reduced by the presence of increased cellular expression of its ligands, CD200 and sCD200. Akman-Karakas et al. showed that in patients with psoriasis, the level of sCD200 was higher compared to healthy controls. Similar results were described by Li et al. in patients with SLE. They detected elevated levels of sCD200 and decreased expression of CD200R1 [42]. The decreased expression of CD200R1 indicates a pro-inflammatory state. On the other hand, sCD200R1 has pro-inflammatory properties, and its reduction, to a certain level, could be beneficial. From this perspective, our results are consistent with the current knowledge addressing reduced expression of CD200R1 and increased expression of CD200 in chronic diseases.

CD5L is involved in a wide variety of processes in organisms. Its expression on Th17 cells, which are the crucial player in the pathogenesis of psoriasis and MetS, is necessary to maintain their nonpathogenicity. It also regulates lipid metabolism in adipocytes and monocyte activity, promotes DAMP and PAMP internalization and removal, and decreases the synthesis of pro-inflammatory cytokines. On the other hand, it drives TLR4 activation. Its expression is up-regulated in the inflammatory microenvironment [16,43].

In our study, CD5L expression was significantly higher in MetS patients compared to nonMets patients and MetS controls (*p* < 0.02 and *p* < 0.08). The lowest levels of CD5L were found in MetS controls. However, other studies have demonstrated its elevation and its important role in metabolic syndrome [44].

It is possible that although CD5L plays an important role in the pathogenesis of metabolic syndrome, its expression is not significantly induced by this condition alone. Studies on this topic are lacking. If there is an increase in CD5L expression in obese subjects, it is in combination with another pathology, as in our study or in the study by Shoji et al., who detected significantly higher expression of CD5L in obese patients with knee osteoarthritis compared to lean patients [45].

In other studies, CD5L is a marker of inflammation, and it is also frequently a marker of a worse prognosis of pathology. Elevation of CD5L was documented in patients with systemic lupus erythematosus, ARDS, chronic kidney disease, cardiovascular events, liver fibrosis, etc. [46,47,48,49,50]. Psoriasis is also an inducer of CD5L expression, which reflects severity. Cretu et al. compared CD5L levels in patients with psoriatic arthritis and osteoarthritis. Psoriatic arthritis was characterized by significantly enhanced production of CD5L [51]. The results of another study by Cretu et al. showed that patients with psoriasis and psoriatic arthritis had higher levels of CD5L compared to controls [52]. These results match ours. 

We also analyzed the correlation between CD5L and other markers. As expected, there was a correlation between CD5L levels and BMI in patients in whom the pro-inflammatory effect of CD5L may be more pronounced than its anti-inflammatory effect, due to stimulation of TLR4 by free fatty acids released from adipocytes [41]. Although CD5L levels increase in chronic inflammation, there is evidence that chronic inflammation could decrease CD200R1 expression and, therefore, could also decrease sCD200R1 levels. Thus the negative correlation of CD5L and CD200R1 which we revealed was also expected [33,38,53]. Interestingly, in MetS controls, we identified a correlation between CD5L and age, but not BMI. We assume that this correlation depends on changes associated with aging; changes in the composition and distribution of adipose tissue, as well as a slightly higher level of inflammation, can be observed especially in elderly persons with MetS [54].

The last evaluated marker of sTLR2 is that it has strong anti-inflammatory effects. TLR2 expression is associated with inflammation. Its soluble form originates both by post-translational modification and by proteolytic cleavage from the membrane. In our study, the levels of sTRL2 did not differ between the two groups or the subgroups. However, other studies described changes in the expression of sTLR2 in chronic inflammatory diseases. Kondelkova et al. described lower levels of sTLR2 in patients with psoriasis compared to healthy controls, despite increased expression of TLR2 [55]. The decreased levels of sTLR2 were also found by Zaharieve et al. in patients with diabetes type II, and by Houssen et al. in patients with SLE, compared to healthy controls [56,57]. While chronic inflammation reduces sTLR2 levels, acute inflammation, especially driven by infection, is associated with increased levels of TLR2 and sTLR2. This is the same pattern which we have seen in CD200R1 expression [58,59].

In our study, we identified a negative correlation between CRP and sTLR2 in psoriasis patients with MetS. These participants had higher levels of CRP, and, thus, more active long-term/chronic inflammation. These results might correspond to those of Houssen et al., who also confirmed that sTLR2 levels were negatively correlated with disease severity [57]. Next, we found that sTLR2 levels were positively correlated with sCD200R1 in patients without Mets. We knew that both markers CD200R1 (which may consequently lead to a reduction in sCD20R1) and sTLR2 are lower in patients with psoriasis [38,55].

Due to the lack of data on psoriasis and metabolic syndromes, as well as the parameters we measured, we can only compare our results with those regarding other chronic inflammatory diseases. Our results do not significantly differ from the currently available data. Our results complement and expand our understanding of the pathogenesis of psoriasis and metabolic syndromes.

## 5. Study Limitations

This study expands upon our previous studies, which evaluated other markers of inflammation, providing a more comprehensive insight into psoriasis and metabolic syndromes. It is limited in the number of participants, especially in subgroup analysis (MetS controls: 15 and nonMetS controls: 49; MetS psoriasis: 15 and nonMetS psoriasis 28), due to availability of participants who meet the specified criteria, logistics, and economics. The results should be interpreted carefully. However, despite the small number of participants, the study has sufficient statistical power. 

Another limitation of our study is the lack of data. These parameters are not a frequent target of researchers, and it was not possible to compare our results with other researchers. 

## 6. Conclusions

Changes in the expression and correlation of the evaluated parameters provide deeper insight into the pathogenesis of inflammation associated with psoriasis and metabolic syndromes. In our study, we measured inflammatory and anti-inflammatory markers associated with psoriasis and the metabolic syndromes sTLR2, CD5L, and sCD200R1, the levels of which have not been evaluated yet. The results showed that psoriasis, metabolic syndromes, and their combination are associated with chronic inflammation, the intensity of which is higher compared to healthy controls. The results further suggest that not only activation of the immune system, but also disruption of inhibitory mechanisms, are involved in the pathogenesis of both diseases. Finally, the selected markers do not seem to be suitable for clinical use to diagnose and monitor disease activity.

## Figures and Tables

**Table 1:** Demographic data.

	Controls (n = 64; Median, Q1–Q3/Numbers)	Patients (n = 43; Median, Q1–Q3/Numbers)
Age (years)	43 (31–57)	50 (34.5–57.8)
nonMetS	49 (77.8%)	28 (65.1%)
MetS	15 (22.2%)	15 (34.9%)
nonSmokers	54 (84.4%)	19 (44.2%)
Smokers	10 (15.6%)	24 (55.8%)
Sex	34 men (53.1%), 30 women (46.9%)	18 men (41.9%), 25 women (58.1%)

**Table 2:** BMI of all participants.

BMI	Median	Q1	Q3	*p* Value
Controls (C; n= 64)	25.7	23.6	28.7	*p* < 0.005
Patients (P; n = 43)	29.3	24.3	32.5	
C nonMetS (n = 49)	25.4	23.7	28.4	NS
C MetS (n = 15)	28.4	23.2	30.6	
P nonMetS (n = 28)	25.3	20.3	30.8	*p* < 0.001
P MetS (n = 15)	32.2	30.1	36.1	

Legend: NS; nonsignificant.

**Table 3:** PASI score of all patients.

PASI	Median	Q1	Q3	*p* Value
P nonMetS (n = 28)	19.4	15.6	26.6	NS
P MetS (n = 15)	18	13.7	25.8	

Legend: NS; nonsignificant.

**Table 4:** The levels of selected markers in controls and patients.

Parameters	Median (Q1–Q3)	*p* Value
CRP		
Controls	2.48 (2.1–3.41)	*p* < 0.05
Patients	3.43 (2.28–5.14)	
sCD200R1 (pg/mL)		
Controls	1314.5 (945–1567)	NS
Patients	966 (506–2164)	
CD5L (ng/mL)		
Controls	1318.8 (928–1937.2)	NS
Patients	1675.9 (1178–2175.8)	
sTLR2 (ng/mL)		
Controls	0.52 (0.41–0.73)	NS
Patients	0.53 (0.45–0.81)	

Legend: NS; nonsignificant.

**Table 5:** The levels of selected markers in subgroups: controls without and with MetS; patients without and with MetS.

Parameters	Median	Q1	Q3	*p* Value
CRP				
C MetS n = 15	2.77	1.92	4.04	NS
C nonMetS n = 49	2.35	2.1	3.38	
P MetS = 15	4.96	3.49	6.27	*p* < 0.01
P nonMetS n = 28	2.68	1.95	4.53	
C MetS n = 15	2.77	1.92	4.04	*p* < 0.05
P MetS = 15	4.96	3.49	6.27	
sCD200R1 (pg/mL)				
C MetS	1305	945	1460	NS
C nonMets	1321	913	1575	
P MetS	1011	521	2120	NS
P nonMetS	947	505.25	2384.5	
CD5L (ng/mL)				
C MetS	1207.1	686	1937.2	NS
C nonMetS	1319.8	1013.3	1951.3	
P MetS	1915	1244	2704	*p* < 0.02
P nonMetS	1400.5	1008.5	2040	
C MetS	1207.1	686	1937.2	*p* < 0.08
P MetS	1915	1244	2704	
sTLR2 (ng/mL)				
C MetS	0.5	0.43	0.69	NS
C nonMetS	0.52	0.4	0.74	
P MetS	0.53	0.47	0.84	NS
P nonMetS	0.49	0.45	0.8	

Legend: NS; nonsignificant.

## Data Availability

The data supporting this article are available upon request to the corresponding author. The data are not publicly available due to maintaining a high level of privacy for the study subjects.
